# Rubinstein-Taybi Syndrome: spectrum of *CREBBP *mutations in Italian patients

**DOI:** 10.1186/1471-2350-7-77

**Published:** 2006-10-19

**Authors:** Angela Bentivegna, Donatella Milani, Cristina Gervasini, Paola Castronovo, Federica Mottadelli, Stefano Manzini, Patrizia Colapietro, Lucio Giordano, Francesca Atzeri, Maria T Divizia, Maria L Giovannucci Uzielli, Giovanni Neri, Maria F Bedeschi, Francesca Faravelli, Angelo Selicorni, Lidia Larizza

**Affiliations:** 1 Division of Medical Genetics, San Paolo School of Medicine, University of Milan, Italy; 2 I Clinica Pediatrica, Fondazione Ospedale Maggiore Policlinico Mangiagalli e Regina Elena, Milan, Italy; 3 Department of Biology and Genetics, Medical Faculty, University of Milan, Milan, Italy; 4 Neuropsichiatria Infantile, Spedali Civili, Brescia, Italy; 5 Laboratory of Molecular Genetics, G. Gaslini Institute, Genova, Italy; 6 Department of Paediatrics, Genetics Unit – Children's Hospital A. Meyer, Florence, Italy; 7 Institute of Medical Genetics, Catholic University, Rome, Italy; 8 Servizio di Genetica Medica, Fondazione Ospedale Maggiore Policlinico Mangiagalli e Regina Elena, Milan, Italy; 9 Department of Human Genetics of Galliera Hospital, Genoa, Italy

## Abstract

**Background:**

Rubinstein-Taybi Syndrome (RSTS, MIM 180849) is a rare congenital disorder characterized by mental and growth retardation, broad and duplicated distal phalanges of thumbs and halluces, facial dysmorphisms and increased risk of tumors. RSTS is caused by chromosomal rearrangements and point mutations in one copy of the CREB-binding protein gene (*CREBBP *or *CBP*) in 16p13.3. To date mutations in *CREBBP *have been reported in 56.6% of RSTS patients and an average figure of 10% has ascribed to deletions.

**Methods:**

Our study is based on the mutation analysis of *CREBBP *in 31 Italian RSTS patients using segregation analysis of intragenic microsatellites, BAC FISH and direct sequencing of PCR and RT-PCR fragments.

**Results:**

We identified a total of five deletions, two of the entire gene and three, all in a mosaic condition, involving either the 5' or the 3' region. By direct sequencing a total of 14 de novo mutations were identified: 10 truncating (5 frameshift and 5 nonsense), one splice site, and three novel missense mutations. Two of the latter affect the HAT domain, while one maps within the conserved nuclear receptor binding of (aa 1–170) and will probably destroy a Nuclear Localization Signal. Identification of the p.Asn1978Ser in the healthy mother of a patient also carrying a de novo frameshift mutation, questions the pathogenetic significance of the missense change reported as recurrent mutation. Thirteen additional polymorphisms, three as of yet unreported, were also detected.

**Conclusion:**

A high detection rate (61.3%) of mutations is confirmed by this Italian study which also attests one of the highest microdeletion rate (16%) documented so far.

## Background

Rubinstein-Taybi syndrome (RSTS; MIM 180849) is a congenital disorder characterized by growth retardation and psychomotor developmental delay, broad and duplicated distal phalanges of thumbs and halluces, and a wide range of typical dysmorphic features. Facial dysmorphology includes down-slanted palpebral fissures, broad nasal bridge, beaked nose and micrognathia [1]. In addition, patients with RSTS have an increased, although not well documented, risk of tumor formation [2,3]. RSTS was shown to be associated with microdeletions and point mutations in the gene encoding the CREB-binding protein (CREBBP, also known as "CBP"), located in 16p13.3 [4]. CREBBP is a transcriptional coactivator and possesses acetyltransferase activity on lysine residues of histones and nonhistone proteins [5]. CREBBP partecipates in basic cellular functions, including growth, differentiation, DNA repair and apoptosis, regulating the expression of many genes [6]. The mutations found in RSTS patients vary from point mutations to relatively large microdeletions, which remove the entire gene, attesting that haploinsufficiency of this dosage-sensitive gene is the ultimate cause of the syndrome. More precisely, studies of patients with missense and splice-site mutations that affect only the histone acetyl transferase (HAT) domain of CREBBP demonstrated that loss of HAT activity is sufficient to cause the syndrome [7,8]. The related p300 protein, which is encoded by the *EP300 *gene on chromosome 22q13.2, shares homology with CREBBP and accordingly serves as a transcriptional coactivator endowed with a HAT domain [9]. Mutations of EP300 have been recently reported in a small subset (3.3%) of RSTS patients [10]. This figure and the low detection rates (from 40% to 56.6%) of *CREBBP *gene assessed by all mutations studies of RSTS patients suggest that other candidate genes might contribute to the pathogenesis of this syndrome [4,10-12]. We recruited 31 patients with consistent RSTS clinical diagnosis and we tested them by microsatellite segregation analysis, FISH, RT-PCR and direct sequencing to find mutations of the *CREBBP *gene. This multimethod approach allowed us to identify five deletions and 14 point mutations, accounting for the highest (61.3%) mutation rate so far assessed. Particularly FISH analysis was instrumental for detecting even subtle microdeletions proving to be a method suitable more than mutation analysis to disclose low level mosaic conditions.

## Methods

### Subjects

We analysed samples of 31 unrelated patients sent to our laboratory with parental consent with the request of genetic analysis for Rubinstein-Taybi syndrome. Studies and procedures were in accordance with the ethical standards of the host institutions. The group consisted of 16 female and 15 male patients, aged 2 to 42 years. Clinical diagnoses were established by different Centers and eventually confirmed by the coordinating clinical Center (Dept of Pediatrics, University of Milan) based on clinical records and photographs, when no pathogenetic mutation could be found. Careful examination of the patients' phenotypes was done, and the presence of malformations and medical complications were confirmed or excluded.

Thirty subjects had a normal karyotype by Giemsa-trypsin banding at 450-band resolution; patient 38 showed a 46, XY, der(14)t(9;14)(p11.2;p11.2) karyotype.

### Cell cultures, chromosome and interphase nuclei preparations

Phytohemagglutinin-stimulated peripheral blood lymphocytes were set up in culture from samples using Chromosome Kit "Synchro" (Celbio) and modified RPMI (Irvine Scientific) plus 5% fetal calf serum (Gibco). The cultures were blocked with colchicine after 72 h. Chromosome preparations were obtained using a standard technique.

### Clone preparations

BAC clones RP11 95J11, RP11 461A8 and RP11 566K11 were supplied by Mariano Rocchi [13]; BAC RP11 1072J2 was purchased from the CHORI BAC PAC Resource Center (Oakland, USA). DNA was obtained starting from a single colony grown in 4 ml of LB medium supplemented with 20 μg/ml chloramphenicol (Sigma) following standard procedures.

### FISH

BAC clones were labelled with digoxigenin-dUTP (Roche Diagnostic) using a nick translation kit (Roche Diagnostic). The FISH experiments were performed according to standard procedures [14]. The chromosomes were counterstained with DAPI in antifade (Vectashild), and then visualised using a Leitz DM-RB microscope equipped for DAPI and FITC/TRITC epifluorescence optics. The images were captured by means of a CCD camera (Hamamatsu 3CCD Camera, C5810) and visualised using Highfish software (Casti Imaging).

### Analysis of FISH signals in mosaic condition

Almost 50 metaphases and 100 nuclei were scored for each sample in order to establish the mosaic condition. Only intact and undamaged nuclei free of cytoplasm were analyzed. Nuclei with low signal intensities, diffuse signals, or absence of signals on both homolog chromosomes were considered to be hybridization failures and were not scored. Two small focal (or paired) signals of the same color and the same intensity, separated by a distance of less than the area of one signal, were considered to be a split signal from one chromosome. Interphase nuclei with one large signal of the same color and increased intensity of fluorescence with the absence of a second hybridization signal in an interphase nucleus were considered to be overlapping (or over-position) of two signals and were not scored. Informative mosaic samples were defined as those in which more than 5% of the nuclei had a reproducible abnormal pattern of signals different from normal chromosomal signals.

### Microsatellite segregation analysis

Fluorescent genotyping was performed as previously described [11]. The following fluorescently dye-labeled chromosome 16p microsatellite markers: MS4, MS2 and D16S3065 (from centromere to telomere) were used for segregation analysis from parents to probands. Fluorescence detection was performed on an ABI 3100 sequencer. ABI PRISM software (Genescan) was used for gel analysis.

### RT-PCR analysis

RT-PCR analysis was performed as previously described [11] for patients 14, 16, 20, 25, 28 and 29 for whom mRNA was available.

### Molecular analysis

The DNA sequencing of all coding exons of the *CREBBP *gene was performed as previously described [11]. Twentysix *CREBBP *PCR genomic fragments were amplified (see Additional file 1). The DNA sequencing protocol was updated for use with the BigDye Chemistry, Versions 2.0 and 3.1, and the ABI PRISM 310 and 377 Genetic analyzer systems (Applied Biosystems, Darmstadt, Germany). If a mutation or polymorphism was found, a second sequencing run was performed for confirmation. The origin of the mutation or polymorphism, whether *de novo *or familial, was established if parental DNA was available. Paternity testing was not performed due to legal restrictions. The Human Gene Mutation Database (HGMD) [15], recent publications [8,10-12], and the dbSNP database [16] were consulted to check whether sequence variations were previously reported or not. Missense mutations of patients 15 and 46 identified in this study were absent in 100 controls, while that carried by patient 23 was absent in 50 controls.

## Results

### Clinical evaluation

**Figure 1:**
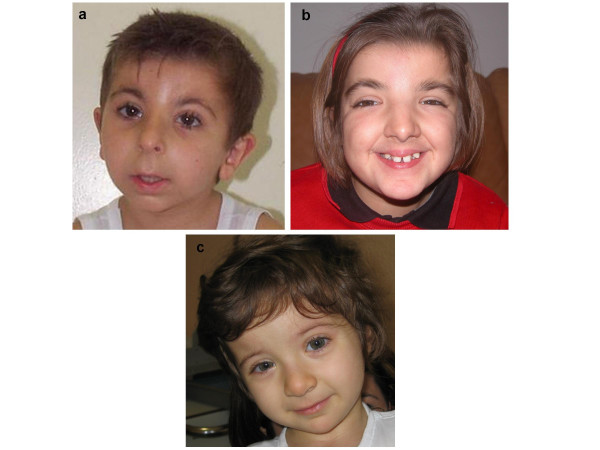
**a **Patient 16 (negative for *CREBBP *mutations); **b **patient 17 (c.3715_3716delAA in *CREBBP *gene); **c **patient 46 (c.4627G>T in *CREBBP *gene). The typical RSTS facial features are visible in the three patients.

Thirty-one Italian RSTS patients, whose main signs are reported in Table 1, were carefully clinically analysed. Sex ratio was F:M = 16:15. All patients had psychomotor/mental retardation, which ranged from mild to severe in 17 cases. Microcephaly was evident in 20 (64.5%); a subset (12 cases) accounting for 61.3% of RSTS patients manifested poor growth. The main facial features were specifically described and/or evident from photos (see Figure 1) and the overall facial gestalt was absolutely typical in all but one patient. As reported in Table 1, the typical large thumb was evident in 93% (27/29 patients), and in 11 cases it was also abducted; the hallux was large in 89% (26/29), and only in 4 cases it was valgus/varus. Only 11 individuals had major malformations of internal organs or limbs: congenital heart disease (patent ductus arteriosus, ventricular/atrial septum defects, bicuspid aorta) was reported in 6 of them, cryptorchid testes in 3, coloboma of the optic nerve, renal hypoplasia, duodenal malrotation and hexadactyly in one. In 5 patients supernumerary nipples were evident. Only in two cases (pt 16 and 17) lacrimal ducts stenosis and sensorineural deafness (pt 14 and 38) were signaled. In case 46 the presence of multiple epitheliomas was reported. In another single case (pt 15) Perthe's disease was found.

**Table 1:** Main clinical signs of the thirty-one Italian RSTS patients.

patient	age	sex	microcephaly	facial features	PMR/MR	large/bifid abducted thumb	hallux large/bifid	hallux valgus/varus	poor growth	malformations	CREBBP mutation
46*	3	F	-	+	+ moderate	+/-	+	-/-	+	+	missense
42	18	F	+	+	+	+/+	+	-/-	+	-	deletion
32	4	F	+	+	+	+/-	+	+/-	+	-	nonsense
34	2	M	+	+	+ moderate	+/-	-	-/-	+	-	nonsense
15	11	M	+	+	+ severe	+/-	+	-/-	+	+	missense
2	10	F	+	+	+ moderate	+/-	+	-/-	+	-	insertion
11	14	M	+	+	+	+/+	+	-/-	+	+	nonsense
17	14	F	+	+	+	+/-	+	-/-	+	+	deletion
20	10	F	+	+	+ severe	+/-	+	-/+	+	-	splice-site mutation
21	7	M	+	+	+	+/+	+	+/+	-	+	deletion
22	30	F	+	+	+	+/+	+	-/-	+	-	deletion
23	5	F	-	+	+ moderate	+/+	+	-/-	-	+	missense
24	7	F	-	+	+	+/-	+	-/-	+	-	nonsense
35	39	M	-	+	+	+/-	+	-/-	+	-	nonsense
40	26	F	+	nd	+ severe	+/-	+	nd	+	-	mosaic del 5'
66	6	M	-	+	+ severe	+/-	+	-	-	+	mosaic del 5'
41	25	F	+	+	+	+/+	+	-/-	+	-	del mat
38	5	M	-	+	+ severe	-/-	+	-/-	-	-	mosaic del 3'
30	26	F	+	+	+	+/+	+	-/-	+	-	del mat
36	11	F	-	+	+ borderline	+/-	-	-/-	-	-	neg
39	13	M	+	+	+	-/-	+	-/-	+	+	neg
43	11	M	+	+	+ servere	nd	nd	nd	-	nd	neg
44	21	M	nd	+	+	+/+	+	-/-	-	-	neg
1	9	M	+	+	+ moderate	+/-	-	-/-	-	-	neg
12	17	M	+	+/-	+ moderate	+/-	+	-/-	+	-	neg
14	6	F	-	+	+ moderate	+/+	+	-/-	-	+	neg
16	8	M	+	+	+ moderate	+/+	+	-/+	+	+	neg
25	4	M	-	+	+ mild	+/-	+	-/-	-	+	neg
45	28	F	nd	+	+ moderate	nd	nd	nd	+	nd	neg
28	42	F	+	+	+	+/+	+	-/-	-	-	neg
29	37	M	+	+	+	+/-	+	-/-	-	-	neg

*development of multiple epiteliomas

### Mutation analysis

**Figure 2:**
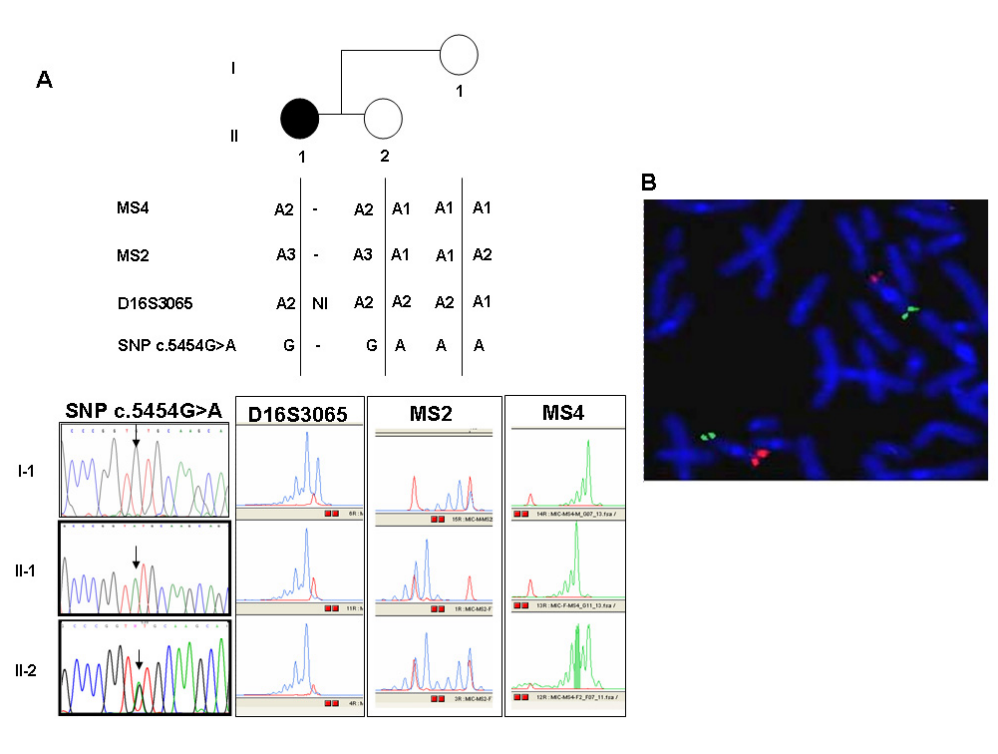
*CREBBP *deletion in patient 30 detected through: **a **genotyping of intragenic polymorphic markers (MS4, MS2, D16S3065 and SNP c.5454G>A) in the available family members allowing haplotype reconstruction; **b **Double color FISH analysis showing a signal of highly reduced intensity on one chromosome 16p for BAC 1072J2, specific for *CREBBP *(in red). The residual hybridization is accounted for by the size of the genomic clone higher than the deleted region. The control subtelomeric 16q BAC 566K11 shows a comparable signal on both chromosomes (in green).

**Figure 3:**
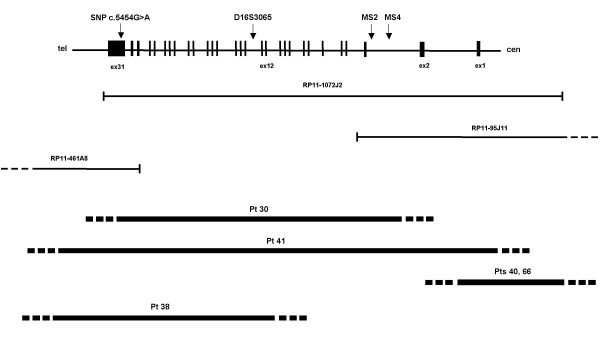
Mapping of the five deletions on the *CREBBP *genomic region. Top: genomic organization of *CREBBP *with exons represented by black boxes and introns as connecting lines. The intragenic polymorphic loci are positioned across the gene. Middle: BAC clones used for preliminary FISH analysis, indicated by bars. Bottom: deletions are shown by thick lines; deletion ends containing the breakpoints, are represented by dashed lines.

We screened the 31 recruited patients for point mutations, small deletions or insertions, and large deletions and duplications of the *CREBBP *gene. First, by genotyping the polymorphic intragenic repeats MS4, MS2 and D16S3065 [11] plus one SNP (c.5454G>A, [12]) we could identify two deletions spanning the whole gene in patients 30 and 41. Both deletions affected the maternal chromosome. Figure 2 shows for patient 30: (A) the haplotype reconstruction attesting the lack of three maternally contributed intragenic polymorphic loci; (B) FISH of the *CREBBP *spanning BAC 1072J2 evidencing a signal of reduced intensity on one chromosome 16. By means of FISH analysis we detected three additional deletions, all in a mosaic condition. Two (pt 40, pt 66) affect the 5' of the gene, while one (pt 38), spans mainly the central region of the gene and its flanking 3' sequences. The last one is present in a structurally rearranged karyotype (see *subjects *in the methods section). The mosaic condition has been confirmed in two patients from which buccal smears were available. Scoring of *CREBBP *specific signals on both lymphocytes (> 150 cells- metaphases and nuclei) and buccal smears (>100 nuclei) gave comparable results in showing an average 20% of deleted cells in both cases. A fraction of 30% deleted cells was estimated on the third case for whom a sample of 300 cells from peripheral blood was scored. Current studies are in progress aimed to evaluating the extent and boundaries of all the deletions. These data will be reported elsewhere. A schematic view of the identified deletions mapped on the encompassed genomic *CREBBP *region is shown in Figure 3.

**Figure 4:**
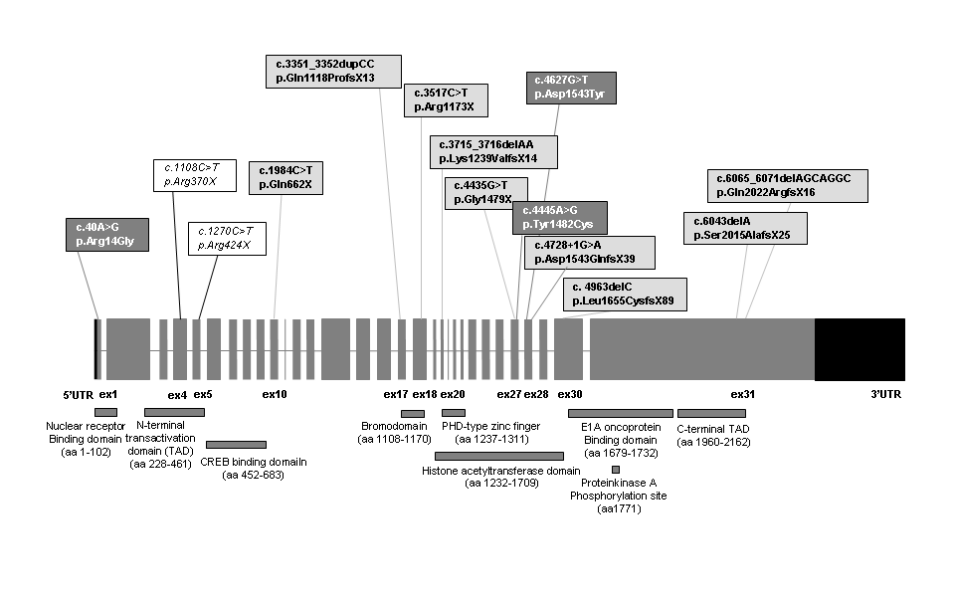
Location of the 14 causative CREBBP mutations found in this study. Only exons are drawn to scale. The histone acetyl transferase domain and the plant homeodomain (PHD)-type zinc finger are indicated according to Kalkhoven et al. (2003), other domains according to Giles et al. (1997). Truncating mutations are in light-grey, missense mutations in dark-grey and the two previously reported mutations in italics.

Then we screened the remaining 26 patients for point mutations by direct sequencing of 26 PCR genomic fragments encompassing the complete coding sequence and splice sites of *CREBBP*. We identified 14 sequence alterations of pathogenic significance: all are listed in Table 2 and shown in Figure 4 in their location within exons encoding different CREBBP domains. Although the identified mutations are distributed across the whole gene without apparent hot-spots, six, including two missense (p.Tyr1482Cys; p.Asp1543Tyr), and four truncating mutations (p.Lys1239ValfsX14; p.Gly1479X; p.Asp1543GlnfsX39; p.Pro1655CysfsX89) are clustered within the HAT domain. Out of the remaining mutations, which map outside the HAT domain, five of eight are predicted to lead to premature translation stops upstream of the HAT region, thus indirectly spoiling its function. They comprise p.Arg370X and p.Arg424X in the N-Terminal Transactivation Domain (TAD), p.Gln662X in the CREB Binding Domain and p.Gln1118ProfsX13 and p.Arg1173X in the Bromodomain. The remaining three mutations, p.Arg14Gly in the 5' Nuclear Receptor Binding Domain, p.Ser2015AlafsX25 and p.Gln2022ArgfsX16 in the C-Terminal TAD, do not affect the HAT domain at all.

**Table 2:** Fourteen causative CREBBP mutations detected by direct sequencing.

PATIENT	MUTATION	TYPE
23	Ex 1: c.40 A>G – p.Arg14Gly	missense
24	*Ex 4: c.1108C>T – p.Arg370X (Bartsch et al.2002)*	nonsense
35	*Ex 5: c.1270C>T – p.Arg424X (Bartsch et al.2005)*	nonsense
11	Ex 10: c.1984C>T – p.Gln662X	nonsense
2	Ex 17: c.3351_3352dupCC – p.Gln1118ProfsX13	insertion
32	Ex 18: c.3517C>T – p.Arg1173X	nonsense
17	Ex 20: c.3715_3716delAA – p.Lys1239ValfsX14	deletion
34	Ex 27: c.4435G>T – p.Gly1479X	nonsense
15	Ex 27: c.4445A>G – p.Tyr1482Cys	missense
46	Ex 28: c.4627G>T – p.Asp1543Tyr	missense
20	Ex 28: c.4728+1G>A	splice-site mutation
22	Ex 30: c.4963delC – p.Leu1655CysfsX89	deletion
42	Ex 31: c.6043delA – p.Ser2015AlafsX25	deletion
21	Ex 31: c.6065_6071delAGCAGGC – p.Gln2022AgfsX16	deletion

In italics: mutations previously described.

**Figure 5:**
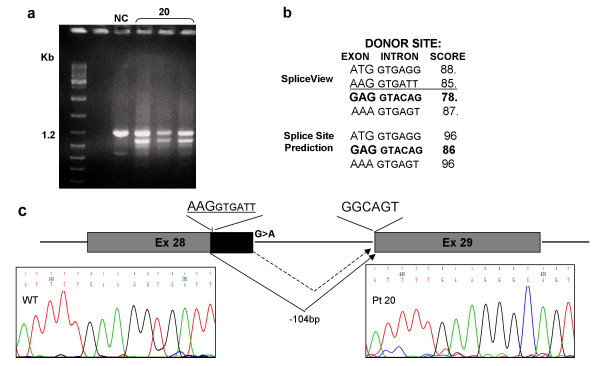
RT-PCR and bioinformatic analysis of the splice site mutation (c.4728+1G>A) of pt 20. **a **RT-PCR of the CREBBP fragment including Ex 28 and 29 evidenced an additional smaller fragment (NC: normal control). **b **bioinformatic analysis with Splice Site Prediction [18] and the SpliceView [19]: the physiological donor site is in bold; the *de-novo *donor site activated is underscored. **c **schematic diagram of the misplicing predicted to occur in this patient with the cryptic donor site underscored. On the left electropherogram of the normal donor site; on the right the electropherogram attesting activation of the cryptic donor site.

Twelve of 14 mutations are novel, while p.Arg370X has been reported by several groups [8,10,11,17] and p.Arg424X has been also recently described [12]. As to the nine different single nucleotide substitutions they consist of five nonsense (pts 24, 35, 11, 32 and 34), three missense (pts 23, 15 and 46) and one splice site mutation (patient 20). Out of the five sequence alterations producing a frameshift in the reading frame, one is a 2 bp-duplication (pt 2) leading to the potential incorporation of 12 anomalous aminoacids, three are deletions sized 1, 2 and 7 nucleotides respectively (pts 22, 17 and 21) predicting 88, 13 and 15 anomalous aminoacids and one is a splice site mutation (c.4728+1G>A) affecting IVS 28 donor site. Analysis of this mutation with two different softwares, the Splice Site Prediction by Neural Network (BDGP) [18] and the SpliceView [19] indicated that the splice donor site was suppressed (predicted scores = 0%) and a novel candidate donor site 260 bp upstream the mutation was activated. The SpliceView software, also detected a near donor site, 104 bp upstream the splice mutation. We were able to identify *CREBBP *transcripts from patient 20 also including a smaller transcript, in addition to the normal one (Figure 5). Direct sequencing of the anomalous transcript confirmed the activation of the nearest upstream cryptic donor site predicted by the SpliceView software. The frameshift produced by missplicing should lead to the incorporation of 38 anomalous aminoacids.

**Figure 6:**
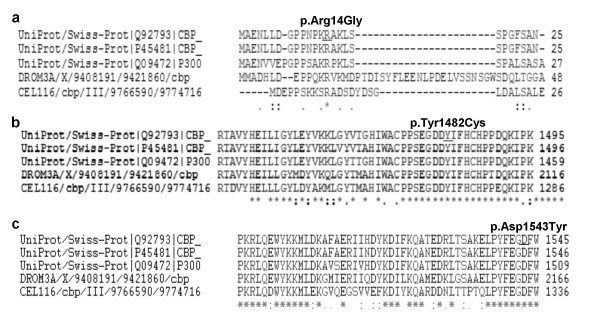
Conservation of amino acids that are predicted by ClustalW [20] to change by three missense mutations identified in this study. **a **p.Arg14Gly of pt 23; **b **p.Tyr1482Cys of pt 15; **c **p.Asp1543Tyr of pt 46. The changed residues are conserved in man (GeneBank: Q92793), in mouse (GeneBank: P45481), in *Drosophila melanogaster *(DROM3A), in *Caenorabditis elegans *(CEL116) and in P300 protein.

**Figure 7:**
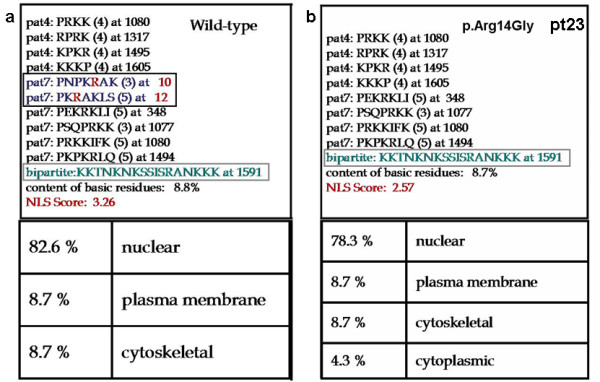
Nuclear Localization signals (NLSs) predicted by PSORTII [21] analysis. **a **wild-type CREBBP with three putative NLSs (two monopartite and one bipartite) evidenced by squares: the affected Arg14 is marked in red; **b **Mutated CREBBP of patient 23 with Arg14Gly substitution with only one predicted NLS. The bottom panels show the cellular localization predictions of wt and mutated proteins.

All three missense changes, p.Arg14Gly (pt 23), p.Asp1543Tyr (pt 46) and p.Tyr1482Cys (pt 15) were not previously reported. They were established to have occurred *de novo *and were found absent in 50 (p.Arg14Gly) and in 100 (p.Asp1543Tyr and p.Tyr1482Cys) healthy individuals, respectively. Furthermore all of them affect aminoacids that are highly conserved both through evolution and in the related p300 (Figure 6) [20]. p.Arg14Gly in the 5' Nuclear Receptor Binding Domain is predicted by PSORTII [21] to cause the destruction of a monopartite Nuclear Localization Sequence (NLS) (Figure 7). This NLS is a typical monopartite cluster of basic residues, with a conserved pattern starting with Pro, followed by Lys or Arg in 3 out of 5 residues (PKRAKLS) [22,23]. In addition the same software predicted another central bipartite Nuclear Localization Signal at 1591 (KKTNKNKSSISRANKKK). Both the NLSs are highly conserved in mouse CREBBP and in p300 protein. p.Tyr1482Cys and the p.Asp1543Tyr are both in the HAT domain. Finally we detected 18 CREBBP polymorphisms, including 12 single nucleotide polymorphisms (SNPs) and a 3'UTR 1 bp-duplication (see Additional file 2). Three of the sequence variations have not been previously reported, including two intronic SNPs (c.3983-22C>T, c.4560+14A>G) and the 3'UTR 7329+25dupC. Conversely the sequence variation (c.5933A>G), was reported in other patients with RSTS [11,12]. This substitution, causing the p.Asn1978Ser change in a conserved glutamine-rich region, downstream the HAT domain, has been inherited by our patient 22 by his apparently normal mother. The same patient was found to bear a *de novo *truncating mutation in the exon upstream the p.Asn1978Ser change (c.4963delC). We were able to establish that both mutations occurred on the maternal allele in a cis configuration (data not shown). Moreover we detected the same nucleotide substitution in one of 50 controls analysed.

## Discussion

By investigating 31 RSTS Italian patients using approaches suitable to detect most types of *CREBBP *mutations we identified *bona fide *causative lesions in 19 subjects (61.3%), a score slightly higher than that recently reported [12]. Complementary application of FISH and microsatellite analysis allowed us to detect five microdeletions, (which refined FISH characterization will be published elsewhere) one of the highest microdeletion rate (16%) documented so far, a finding that emphasizes possible underestimation of *CBP *microdeletions in RSTS patients [24-26]. Data achieved using realtime quantitative PCR are consistent with this view [27].

Interestingly this is the first report describing mosaicism for *CREBBP *deletions, indicating the contribution of mitotic errors to this rare syndrome, that might exploit the same breakpoints of the highly unstable region around intron 2 of *CREBBP *evidenced in cancer [28-30]. It should be noted that in one case (pt 38) the *CREBBP *mosaic deletion is in the context of a rearranged karyotype, which may account for the complex phenotype of the patient.

By sequencing all exons and splice sites of the *CREBBP *gene, we identified on a total of 26 patients 14 different mutations, only two of which have been described [8,10-12,17]. Neither a predominant type of mutation nor clustering of mutations within the *CREBBP *gene are apparent. In agreement with previous studies, most mutations (11/14, >78%) predict premature stop codons [4,7,8,11,12,17,24]. Since RSTS is an autosomal dominant trait, it can be assumed that truncating mutations are disease causing mutations making superfluous to determine their *de novo *origin [10,11]. We also detected three putative missense mutations, in which the pathogenic role is much less clear. However, we were able to confirm the *de novo *origin of all three missense mutations which were found only once among 26 patients analysed and were never observed in a 100 normal controls (50 in the case of patient 23), ruling out that they are unreported CBP polymorphisms. Moreover, when compared with the murine CREBBP (Genbank: S66385) [31], which shows 95% identity across the whole 2441 aa length of the protein, all the three mutated residues were conserved (Figre 6). More generally their conservation, both phylogenetic, from *fruit fly *to *C. Elegans*, and in the related p300 protein highlights the functional importance of these residues in the protein. Interestingly, two missense mutations are in the HAT domain of CREBBP, where similar mutations were shown to be sufficient to cause the RSTS phenotype as they destroyed the acetyltransferase activity [7,8]. Furthermore p.Tyr1482Cys (patient 15) is in the postulated coenzyme A binding site (aminoacids 1459–1541). It may not be fortuitous that the phenotypic presentation of this patient strikingly overlaps the clinical presentation of a second patient (20), carrying a splicing mutation affecting the same domain. Application of RT-PCR allowed to document the deleterious consequence of the splicing mutation and to better interpret the patient's phenotype. Indeed both patients 15 and 20 are affected by severe mental retardation and complete absence of speech. The third missense mutation, p.Arg14Gly, found in patient 23, is localised in the 5' end of the CREBBP protein, where no other missense mutation has been reported. Aminoacids from 1 to 100 are known to be responsible for the Nuclear-Receptors binding (RAR, GR TXR) [32]. Moreover, according to the PSORTII software the resulting aminoacid change is predicted to disrupt a putative Nuclear Localization Signal. Functional studies of this mutant protein are in progress to determine whether the predicted NLS is necessary for the correct import of the protein into the nucleus. It should be noted that patient 23 has a mild phenotype, characterised by normal growth and absence of microcephaly. This peculiar phenotype might be accounted for by a reduced amount of CREBBP protein in the nucleus because of impaired translocation of the mutated product, rather than by a reduced binding to Nuclear-Receptors.

In this study we detected the c.5933A>G leading to p.Asn1978Ser change in patient 22, who carries also a *de novo *truncating mutation (c.4963delC). We were able to establish the cis phase of the two alterations on the maternal allele. The same p.Asn1978Ser change was found in a classic RSTS patient [11] and has been recently described as a recurrent mutation, in a patient displaying an incomplete RSTS phenotype [12]. We detected the same change in our patient 22's healthy mother, and subsequently in one of 50 additional control individuals who were studied: thus we infer it may be counted among CREBBP polymorphisms. Consistent with this view, the resulting amino acid substitution, located outside the HAT domain, affects an aminoacidic residue conserved in the mouse, but not either in *fruit fly *and *C. Elegans*, or in p300 (Figure 6). Interestingly, a different change of the same residue (p.Asn1978Asp) has been recently detected in an ovarian and a breast tumour from unrelated individuals. Mutation analysis in germline DNA which could be performed on one of the two individuals showed the same sequence alteration present in the tumours [33]. The authors emphasised that the individual carrying the mutation in the germline does not have the RSTS phenotype, as expected of someone with a *CREBBP *mutation. All the cumulated evidences indicate the affected *CREBBP *codon as a highly polymorphic site, resulting in a aminoacidic change which does not disrupt protein function sufficiently to cause RSTS or to predispose to breast and/or ovarian cancer. The overall data might help to interpret this sequence change and to keep it into account in genetic counselling.

Finally, we identified 13 additional sequence variations, which can be added to the list of known CREBBP polymorphisms (see Additional file 2). Interestingly our patients 16 and 25 share five different polymorphisms inherited from their parents, in agreement with a recent report [12]. Also our two families do not appear to be related, but extended genetic histories were not available.

## Conclusion

By combining different techniques we identified a *CREBBP *mutation in 61.3% of RSTS cases, a mutation rate higher than that of a recent study [12]. Intragenic microsatellite markers and FISH analysis enabled us to identify one of the highest microdeletion rate (16%) documented so far [24-26,34]. No significant correlation could be established between different types of mutation and the clinical presentation and significantly, the patients proved negative to *CREBBP *mutation screening (both point mutations and deletions) represented the whole RSTS phenotypic spectrum. The overall mutation rate attested by our study and the fact that most *CREBBP*-negative patients did not display a mild or borderline RSTS clinical presentation provide further indirect evidence on the involvement of genes other than *CREBBP *in the RSTS phenotype. Indeed, a few *CREBBP *lesions might have been underestimated, as we ruled out silent exonic alterations affecting splicing enhancers (in the *CREBBP*-negative patients 14, 16, 25, 28 and 29), but not intronic alterations affecting splicing as analysis at the RNA level was precluded. Similarly the *CREBBP *promoter could not be tested, as direct sequencing was attempted, but raised technical problems. However the putatively missed *CREBBP *alterations likely do not cover the whole fraction of negative cases with a consistent RSTS phenotype. It may be that a few of the screened patients will turn out, upon a closer clinical examination, to have a different syndrome that resembles RSTS. However, considering the larger set of RSTS patients cumulatively ascertained by other groups, yet mutations were not found in more than half their patients as well [11,12]. The recent finding of only three mutations of the CREBBP homologue p300 in a series of 91 patients did not prompt us to screen this gene in our much more restricted cohort [10]. However genetic heterogeneity of RSTS patients is documented and lesions in other genes encoding for proteins that interact with CREBBP in various signal transduction pathways, should be considered.

## Competing interests

The author(s) declare that they have no competing interests.

## Authors' contributions

AB: main investigator with a direct and coordinating role in experimental work; drafted the manuscript and approved the final version of it.

DM: involved in the collection and critical review of clinical data

CG, PC, FM: performed laboratory investigation concerning both mutation analysis and FISH screening and helped to draft the manuscript

SM e PC: involved in the set up of the mutation screening

LG, FA, MTD, MLGU, GN, MFB, FF: clinical geneticists providing selected patients with RSTS

AS: supervisor for patients recruitment and clinical diagnosis

LL: involved in design and coordination of the experimental work; drafted the manuscript and approved the final version of it.

All authors read and approved the final manuscript.

## Pre-publication history

The pre-publication history for this paper can be accessed here:



## Supplementary Material

Additional file 1 Additional Table I. Twentysix CREBBP PCR pairs of primer for DNA sequencing of all coding exons of the CREBBP gene, as previously described by Coupry et al. 2002 Click here for file

Additional file 2 Additional Table II. Thirteen CREBBP polymorphisms detected by direct sequencing. The yet unreported variations are bolded. Click here for file
