# Supplementary material for: Rubinstein-Taybi Syndrome: spectrum of CREBBP mutations in Italian patients
Source: BMC Med Genet. 2006 Oct 19;7:77. doi: 10.1186/1471-2350-7-77 (PMC1626071; doi:10.1186/1471-2350-7-77)
Supplement: Additional file 1 — Additional Table I. Twentysix CREBBP PCR pairs of primer for DNA sequencing of all coding exons of the CREBBP gene, as previously described by Coupry et al. 2002 [file 1471-2350-7-77-S1.doc]

| **Exons**  **Additional Table I** Twentysix CREBBP PCR pairs of primer for DNA sequencing of all coding exons of the CREBBP gene, as previously described by Coupry et al. 2002 | **Primer** | **Conditions** | **# cicles** | **DMSO**  **for 25μl** | **MgCl_2_**  **for 25μl** |
| --- | --- | --- | --- | --- | --- |
| Exon 1 | EX1L  EX1R | 30’’ - 30’’ - 17’’  95°C - 65°C - 72°C | 37 | 0.5μl | 0.75 μl |
| Exon 2 | EX2L  EX2R | 30’’ - 30’’ - 30’’  95°C - 55°C - 72°C | 35 | 0.25μl | 0.75μl |
| Exon 3 | EX3L  EX3R | 30’’ – 30’’ - 20’’  95°C – 55°C – 72°C | 35 | 0.25μl | 0.75μl |
| Exon 4 | EX4L  EX4R | 30’’ – 30’’ – 25’’  95°C – 55°C – 72°C | 35 | 0.25μl | 0.75μl |
| Exon 5 | EX5L  EX5R | 30’’ – 30’’ – 25’’  95°C – 60°C – 72°C | 35/37 | 0.25μl | 0.75μl |
| Exon 6 | EX6L  EX6R | 30’’ – 30’’ – 30’’  95°C – 55°C – 72°C | 35 | 0.25μl | 0.75μl |
| **Exons 7+8** | EX7L  EX8R | 30’’ – 30’’ – 30’’  95°C – 57°C – 72°C | 35 | 0.25μl | 0.75μl |
| **Exons 9+10** | EX9L  EX10R | 30’’ – 30’’ – 45’’  95°C – 55°C – 72°C | 35 | 0.25μl | 0.75μl |
| Exon 11 | EX11L  EX11R | 30’’ – 30’’ – 20’’  95°C – 55°C – 72°C | 35 | 0.25μl | 0.75μl |
| **Exons 12+13** | EX12L  EX13R | 30’’ – 30’’ – 50’’  95°C – 56°C – 72°C | 35 | 0.25μl | 0.75μl |
| Exon 14 | EX14L  EX14R | 30’’ – 30’’ – 25’’  95°C – 55°C – 72°C | 35 | 0.25μl | 0.75μl |
| **Exons 15+16** | EX15L  EX16R | 30’’ – 30’’ – 50’’  95°C – 57°C – 72°C | 40 | 0.25μl | 0.70μl |
| Exon 17 | EX17L  EX17R | 30’’ – 30’’ – 20’’  95°C – 55°C – 72°C | 35 | 0.25μl | 0.75μl |
| **Exons 18+19** | EX18L  EX19R | 30’’ – 30’’ – 1’:00’’  95°C – 56°C – 72°C | 35 | 0.25μl | 0.75μl |
| Exon 20 | EX20L  EX20R | 30’’ – 30’’ – 20’’  95°C – 56°C – 72°C | 35 | 0.25μl | 0.5μl |
| Exon 21 | EX21L  EX21R | 30’’ – 30’’ – 20’’  95°C – 55°C – 72°C | 35 | 0.25μl | 0.75μl |
| **Exons 22+23** | EX22L  EX23R | 30’’ – 30’’ – 30’’  95°C – 55°C – 72°C | 35 | 0.25μl | 0.75μl |
| Exon 24 | EX24L  EX24R | 30’’ – 30’’ – 30’’  95°C – 60°C – 72°C | 35 | 0.25μl | 0.75μl |
| **Exon 25+26** | EX25L  EX25R | 30’’ – 30’’ – 30’’  95°C – 55°C – 72°C | 35 | 0.25μl | 0.75μl |
| Exon 27 | EX27L  EX27R | 30’’ – 30’’ – 20’’  95°C – 60°C – 72°C | 35 | 0.25μl | 0.625μl |
| Exon 28 | EX28L  EX28R | 30’’ – 30’’ – 20’’  95°C – 60°C – 72°C | 35 | 0.25μl | 0.75μl |
| Exon 29 | EX29L  EX29R | 30’’ – 30’’ – 15’’  95°C – 60°C – 72°C | 35 | 0.25μl | 0.75μl |
| Exon 30 | EX30L  EX30R | 30’’ – 30’’ – 20’’  95°C – 60°C – 72°C | 35 | 0.25μl | 0.75μl |
| Exon 31-A | EX31-A  EX31-B | 30’’ - 30’’ - 40’’  95°C - 55°C – 72°C | 35 | 0.25μl | 0.75μl |
| Exon 31-B | EX31-L2  EX31-R4 | 30’’ – 30’’ – 40’’  95°C - 55°C – 72°C | 35 | 0.25μl | 0.75μl |
| Exon 31-C | EX31-L4  EX31-R | 30’’ – 30’’ – 40’’  95°C - 55°C – 72°C | 35 | 0.25μl | 0.75μl |
