# Supplementary material for: Rubinstein-Taybi Syndrome: spectrum of CREBBP mutations in Italian patients
Source: BMC Med Genet. 2006 Oct 19;7:77. doi: 10.1186/1471-2350-7-77 (PMC1626071; doi:10.1186/1471-2350-7-77)
Supplement: Additional file 2 — Additional Table II. Thirteen CREBBP polymorphisms detected by direct sequencing. The yet unreported variations are bolded. [file 1471-2350-7-77-S2.doc]

| PATIENT | SEQUENCE CHANGE | LOCATION | TYPE | ORIGIN | dbSNP rs#cluster ID, ref |
| --- | --- | --- | --- | --- | --- |
| 28 | c.459G>A | 2 | synonymous | PATERNAL | Bartsch et al.2005 |
| 16 | c.939T>C | 3 | synonymous | PATERNAL | 3025702 |
| 25 | c.939T>C | 3 | synonymous | MATERNAL | 3025702 |
| 16 | c.1574-32G>T | INTRON 5-6 | intronic | PATERNAL | 130025 |
| 25 | c.1574-32G>T | INTRON 5-6 | intronic | MATERNAL | 130025 |
| 16 | c.1651C>A | 7 | p.Leu551Ile | PATERNAL | Bartsch et al.2005 |
| 25 | c.1651C>A | 7 | p.Leu551Ile | MATERNAL | Bartsch et al.2005 |
| 16 | c.3837-8C>T | INTRON 21-22 | splice site | PATERNAL | Bartsch et al.2005 |
| 25 | c.3837-8C>T | INTRON 21-22 | splice site | MATERNAL | Bartsch et al.2005 |
| **20** | **c.3983-22C>T** | **INTRON 23-24** | **intronic** | **ND** | **this work** |
| 16 | c.4280+42C>T | INTRON 25-26 | intronic | PATERNAL | 129967 |
| 25 | c.4280+42C>T | INTRON 25-26 | intronic | MATERNAL | 129967 |
| **20** | **c.4560+14A>G** | **INTRON 27-28** | **intronic** | **ND** | **this work** |
| 30 | c.5454G>A | 31 | synonymous | PATERNAL | Bartsch et al.2005 |
| 22 | c.5933A>G | 31 | p.Asn1978Ser | MATERNAL | Bartsch et al.2005 |
| 25 | c.5988C>T | 31 | synonymous | MATERNAL | Bartsch et al.2005 |
| 25 | c.6621G>A | 31 | synonymous | DE NOVO | Bartsch et al.2005 |
| 25 | **7329+25dupC** | **31** | 3'UTR | MATERNAL | **this work** |

**Additional Table II** thirteen CREBBP polymorphisms detected by direct sequencing. The yet unreported variations are bolded.
